# Supplementary material for: Dynamin-related protein 1 deficiency accelerates lipopolysaccharide-induced acute liver injury and inflammation in mice
Source: Commun Biol. 2021 Jul 21;4:894. doi: 10.1038/s42003-021-02413-6 (PMC8295278; doi:10.1038/s42003-021-02413-6)
Supplement: Supplementary file 1 — Supplementary Information [file 42003_2021_2413_MOESM1_ESM.pdf]

Supplementary Information

Dynamin-related protein 1 deficiency accelerates lipopolysaccharide-induced acute liver injury and inflammation in mice

Lixiang Wang, Xin Li, Yuki Hanada, Nao Hasuzawa, Yoshinori Moriyama, Masatoshi Nomura & Ken Yamamoto

Contents

Supplementary Figure 1. LPS-induced inflammatory response in C57BL/6J mice.

Supplementary Figure 2. LPS-induced inflammatory responses in control and *Drp1*LiKO mice.

Supplementary Figure 3. LPS/ATP treatment-induced inflammatory responses in control and *Drp1*LiKO mice.

Supplementary Figure 4. LPS/ATP treatment-induced inflammatory cell infiltration in control and *Drp1*LiKO mice livers.

Supplementary Figures 5-10. Uncropped western blot images.

Supplementary Table 1. Genes involved in the immune system process.

Supplementary Table 2. Genes involved in mitotic nuclear division.

Supplementary Table 3. Reagents used in this study.

Supplementary Table 4. Antibodies used in this study.

Supplementary Table 5. Real-time PCR primers used in this study.

a

b

c

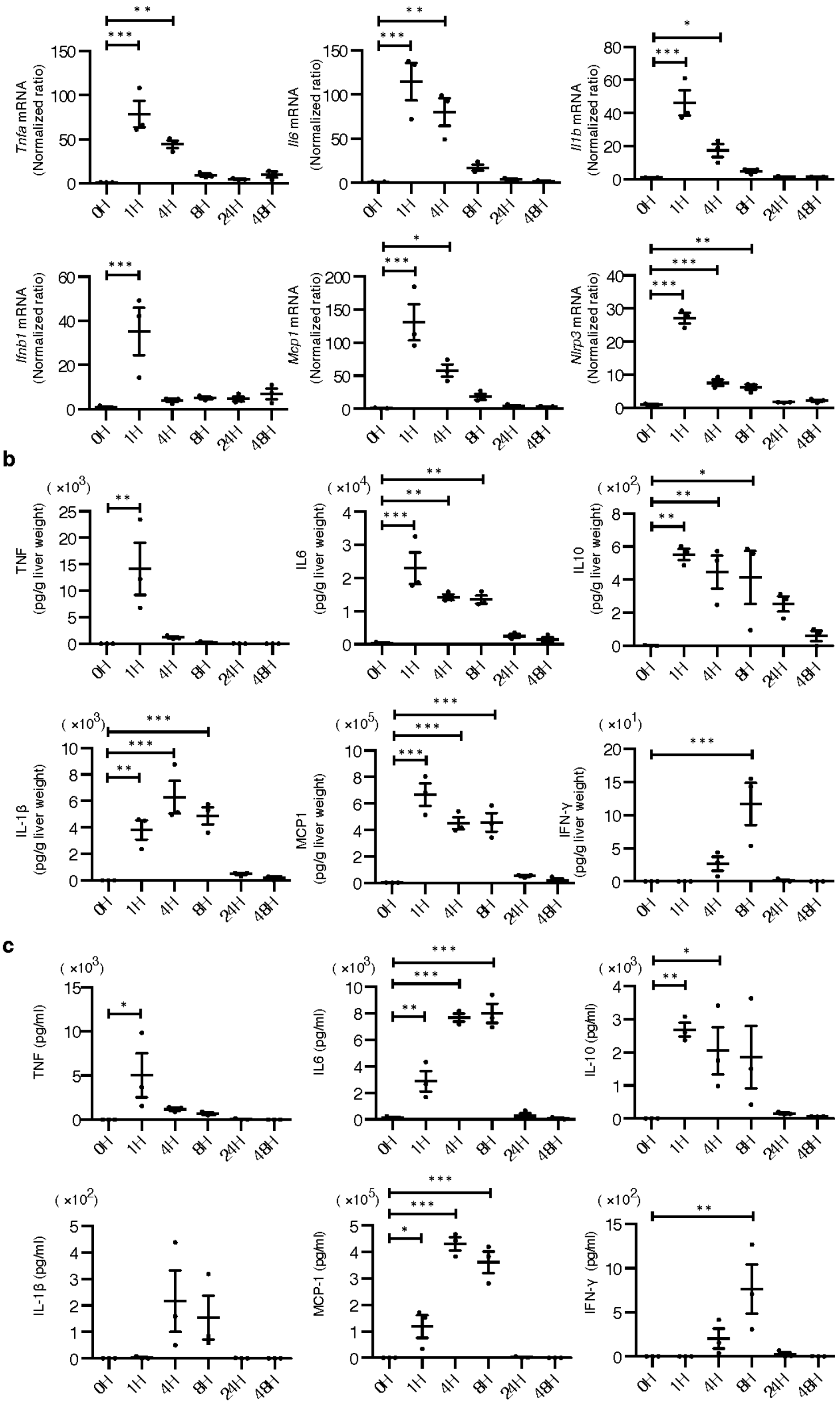

Supplementary Figure 1 Wang et al.

Supplementary Figure 1. LPS-induced inflammatory response in C57BL/6J mice. C57BL/6J mice were treated with saline alone (referred to as LPS 0 h) or LPS (5 mg/kg, IP). (**a**) Gene expression levels of *Tnfa*, *Il6*, *Il1b, Ifnb1*, *Mcp1*, and *Nlrp3* were determined by quantitative real-time PCR analysis of total RNA from saline- or LPS-treated mice livers at indicated time points. Results are normalized to the expression of the *Gapdh* gene and are shown as fold-changes relative to gene expression in saline-treated mice. (**b** and **c**) At the indicated time points after LPS treatment, livers and serum samples were collected. TNF, IL-6, IL-10, IL-1β, MCP1, and IFN-γ levels in the liver (**b**) and serum (**c**) were determined using BD CBA. Values are expressed as mean ± SEM ($n = 3$). *$p < 0.05$, **$p < 0.01$, and ***$p < 0.001$ determined by one-way ANOVA with Dunnett's multiple comparisons test. Abbreviations: LPS, lipopolysaccharide; IP, intraperitoneally; TNF, tumor necrosis factor; IL, interleukin; MCP1, monocyte chemoattractant protein-1; IFN-γ, interferon gamma; CBA, cytometric bead array; PCR, polymerase chain reaction; Gapdh, glyceraldehyde 3-phosphate dehydrogenase; NLRP3, NLR family pyrin domain containing 3; SEM, standard error of the mean.

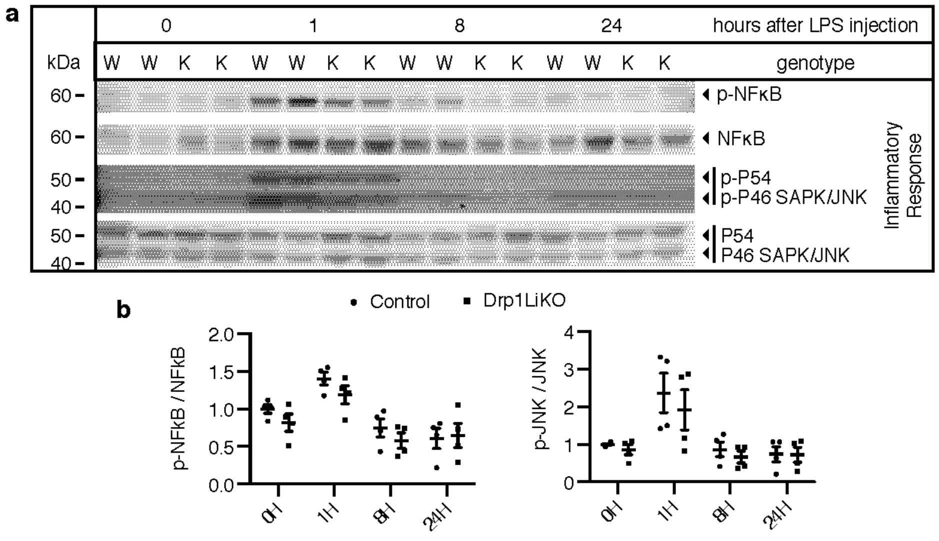

Supplementary Figure 2 Wang et al.

Supplementary Figure 2. LPS-induced inflammatory responses in control and *Drp1*LiKO mice. Control and *Drp1*LiKO mice were treated with saline alone (referred to as LPS 0 h) or LPS (5 mg/kg, IP). (**a** and **b**) At the indicated time points after LPS treatment, livers were collected. p-NF-κB, NF-κB, p-JNK, and JNK protein levels in the liver were determined by western blot analysis. Values are expressed as mean ± SEM (n = 4). *$p < 0.05$ and **$p < 0.01$. Abbreviations: LPS, lipopolysaccharide; *Drp1*LiKO, liver-specific *Drp1*-knockout; IP, intraperitoneally; TNF, tumor necrosis factor; IL, interleukin; MCP1, monocyte chemoattractant protein-1; IFN-γ, interferon gamma; CBA, cytometric bead array; PCR, polymerase chain reaction; SEM, standard error of the mean.

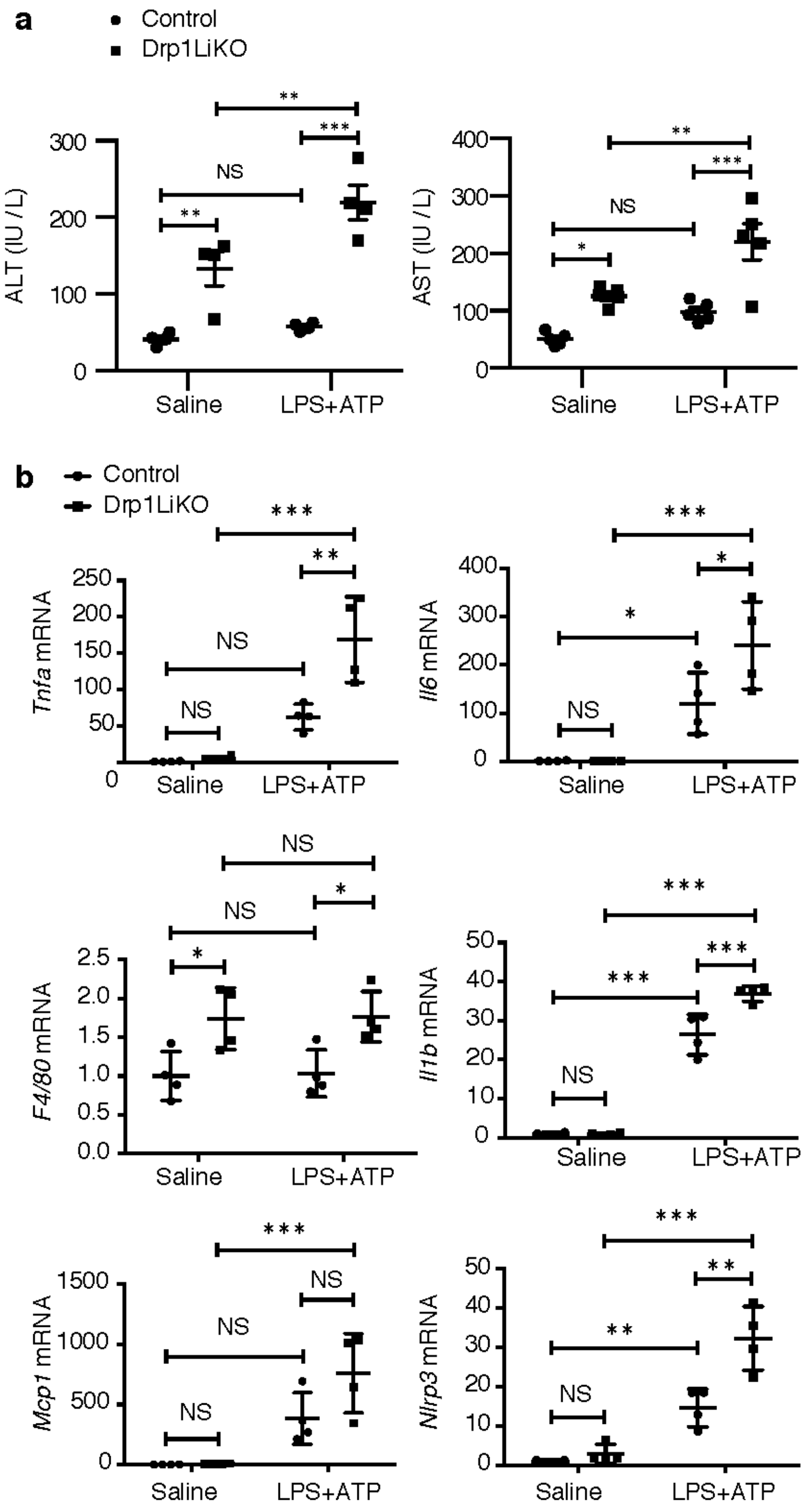

Supplementary Figure 3 Wang et al.

Supplementary Figure 3. LPS/ATP treatment-induced inflammatory responses in control and *Drp1*LiKO mice. Control and *Drp1*LiKO mice were treated with 10-mg/kg body weight LPS (IP) for 4 h followed by an additional 50 µl injection (IP) of 100-mM ATP 30 min before sacrifice. (**a**) Serum ALT and AST values in saline- or LPS/ATP-treated control and *Drp1*LiKO mice. Values are expressed as mean ± SEM (n = 4–5). NS, no significant difference; *$p < 0.05$, **$p < 0.01$, and ***$p < 0.001$ determined by two-way ANOVA with Tukey's post hoc test. (**b**) Gene expression levels of *Tnfa*, *Il6*, *Il1b,* *F4/80*, *Mcp1*, and *Nlrp3* were determined by quantitative real-time PCR analysis of total RNA from saline- or LPS/ATP-treated mouse livers. Results are normalized to the expression of the *Gapdh* gene and are shown as fold-changes relative to gene expression in saline-treated control mice. Values are expressed as mean ± SEM (n = 4). NS, no significant difference; *$p < 0.05$, **$p < 0.01$, and ***$p < 0.001$ determined by two-way ANOVA with Tukey's post hoc test. Abbreviations: LPS, lipopolysaccharide; *Drp1*LiKO, liver-specific *Drp1*-knockout; IP, intraperitoneally; ALT, alanine aminotransferase; AST, aspartate transaminase; *Tnf*, tumor necrosis factor; *Il*, interleukin; *Mcp1*, monocyte chemoattractant protein-1; *Nlrp3*, NLR family pyrin domain containing 3; *Gapdh*,

glyceraldehyde 3-phosphate dehydrogenase; PCR, polymerase chain reaction; SEM, standard error of the mean.

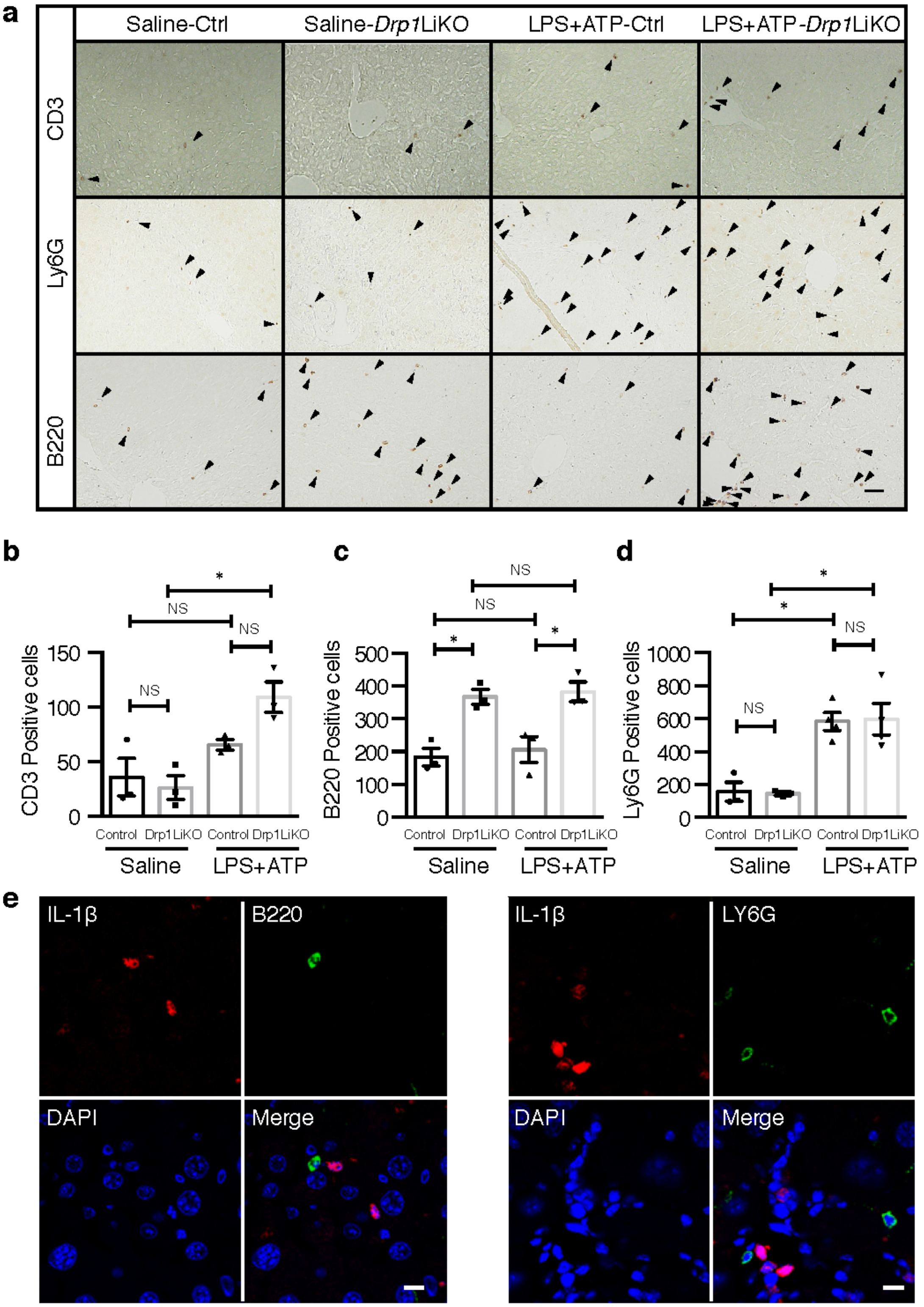

Supplementary Figure 4 Wang et al.

Supplementary Figure 4. LPS/ATP treatment-induced inflammatory cell infiltration in control and *Drp1*LiKO mice livers. (**a–d**) Representative images of CD3, Ly6G, and B220 staining of liver sections from either saline- or LPS/ATP-treated control and *Drp1*LiKO mice. Scale bar = 50 μm. Quantitative analysis of the number of CD3-, Ly6G-, and B220-positive cells was performed by counting cells in 15 high-power fields (20×) per slide from 4 mice per group. Values are expressed as mean ± SEM (n = 4). NS, no significant difference; *p < 0.05 determined by two-way ANOVA with Tukey's post hoc test. (e) Representative images of IL-1β, Ly6G, and B220 staining of livers from LPS/ATP-treated *Drp1*LiKO mice. IL-1β-positive cells were visualized using Alexa Fluor 594-conjugated rabbit anti-goat IgG (red), whereas Ly6G- and B220-positive cells were visualized using Alexa Fluor 488-conjugated goat anti-rat IgG (green). Nuclei were stained with DAPI (blue). Scale bar = 10 μm. Abbreviations: LPS, lipopolysaccharide; ATP, adenosine triphosphate; *Drp1*LiKO, liver-specific *Drp1*-knockout; IP, intraperitoneally; SEM, standard error of the mean; IgG, immunoglobulin G; DAPI, 4′,6-diamidino-2-phenylindole.

Supplementary Figure 5. Uncropped gel pictures for Figure. 2a.

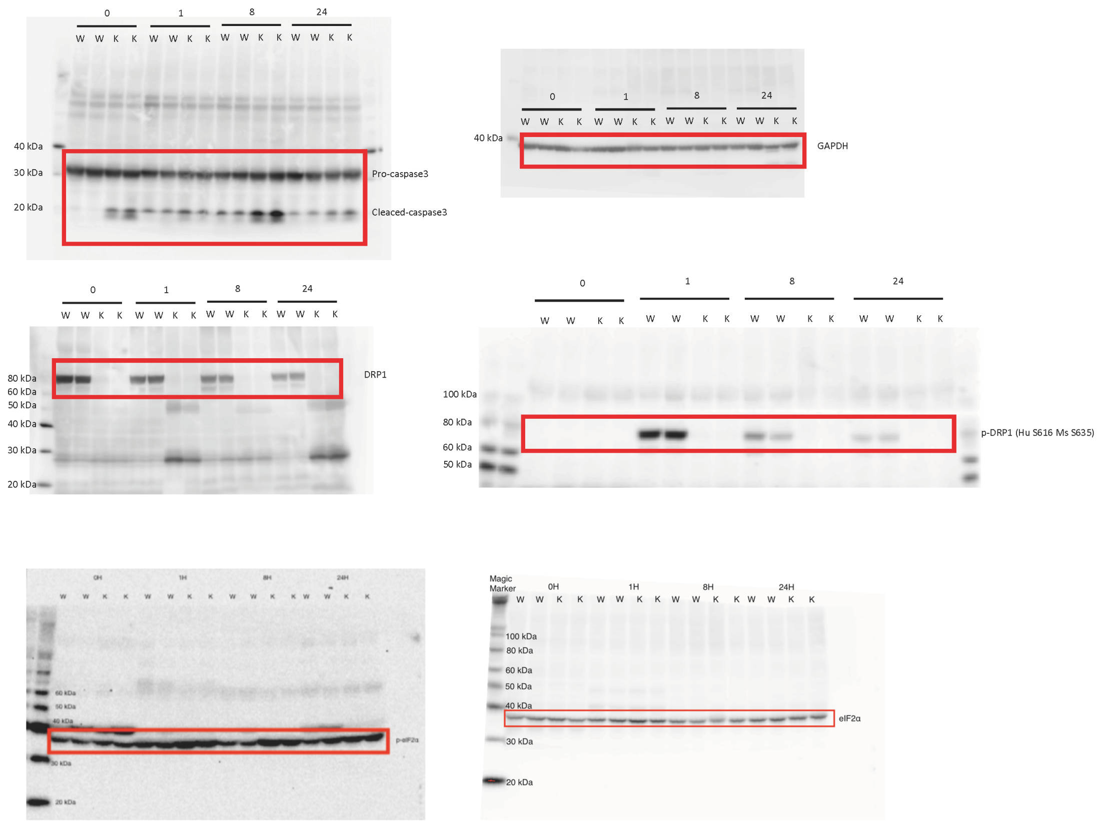

Supplementary Figure 6. Uncropped gel pictures for Figure. 2g.

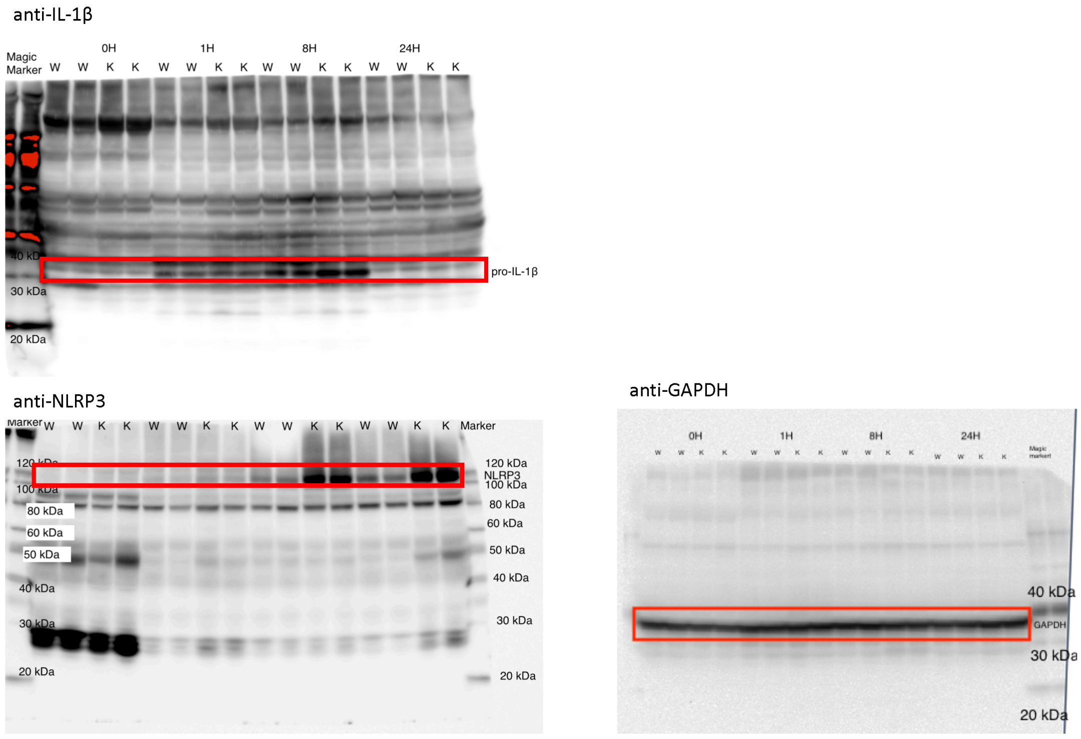

Supplementary Figure 7. Uncropped gel pictures for Figure. 4a.

anti-LC3

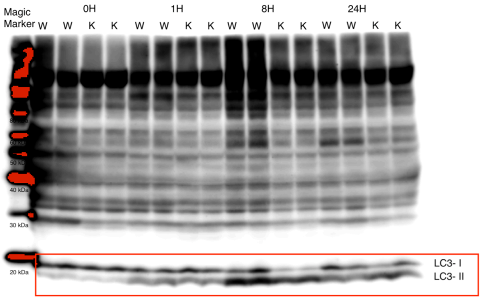

anti-P62

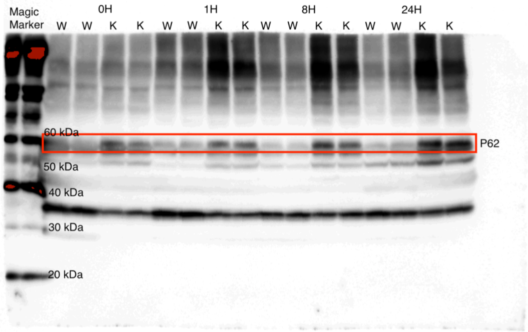

anti-PINK1

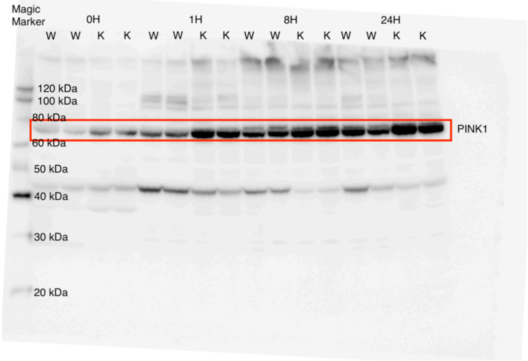

anti-GAPDH

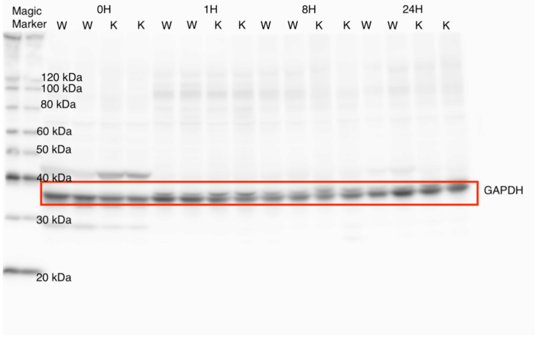

Supplementary Figure 8. Uncropped gel pictures for Figure. 4f.

anti-LC3

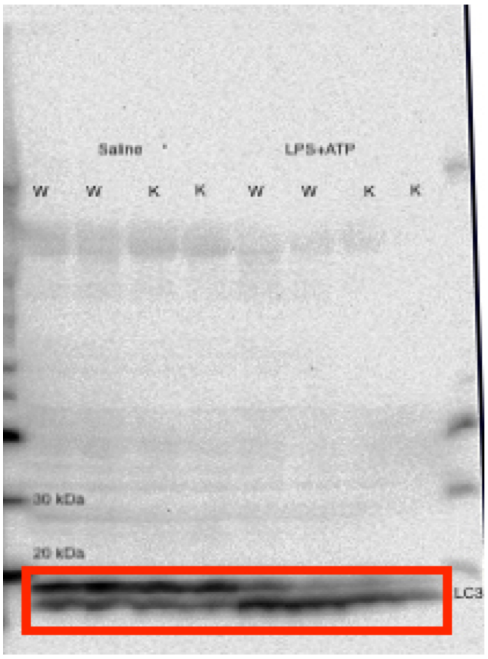

anti-GAPDH

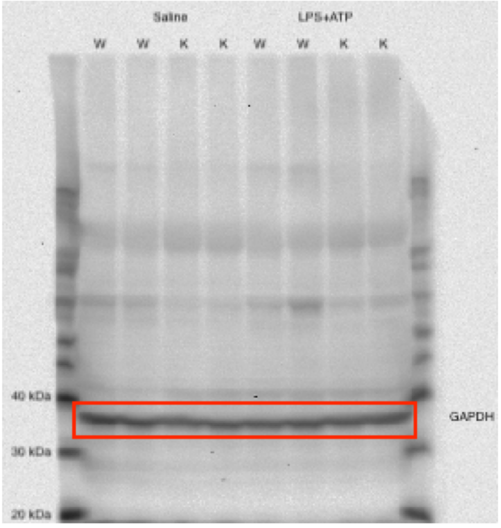

Supplementary Figure 9. Uncropped gel pictures for Figure. 6c.

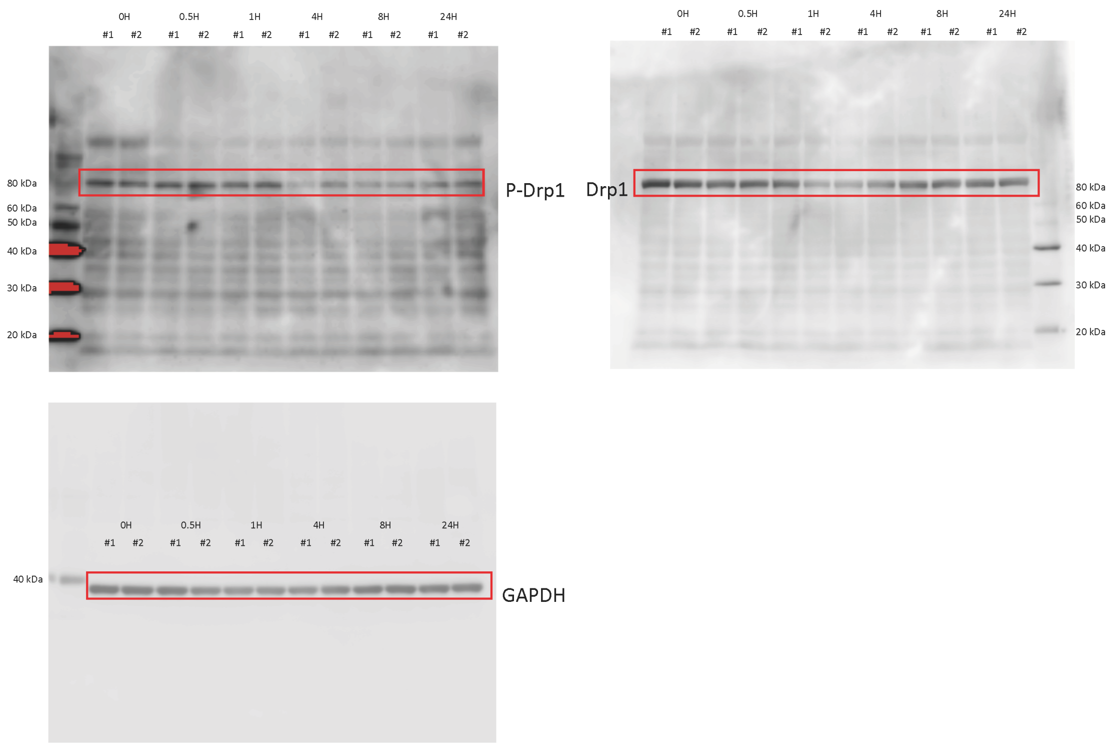

Supplementary Figure 10. Uncropped gel pictures for Supplementary Figure. 2a.

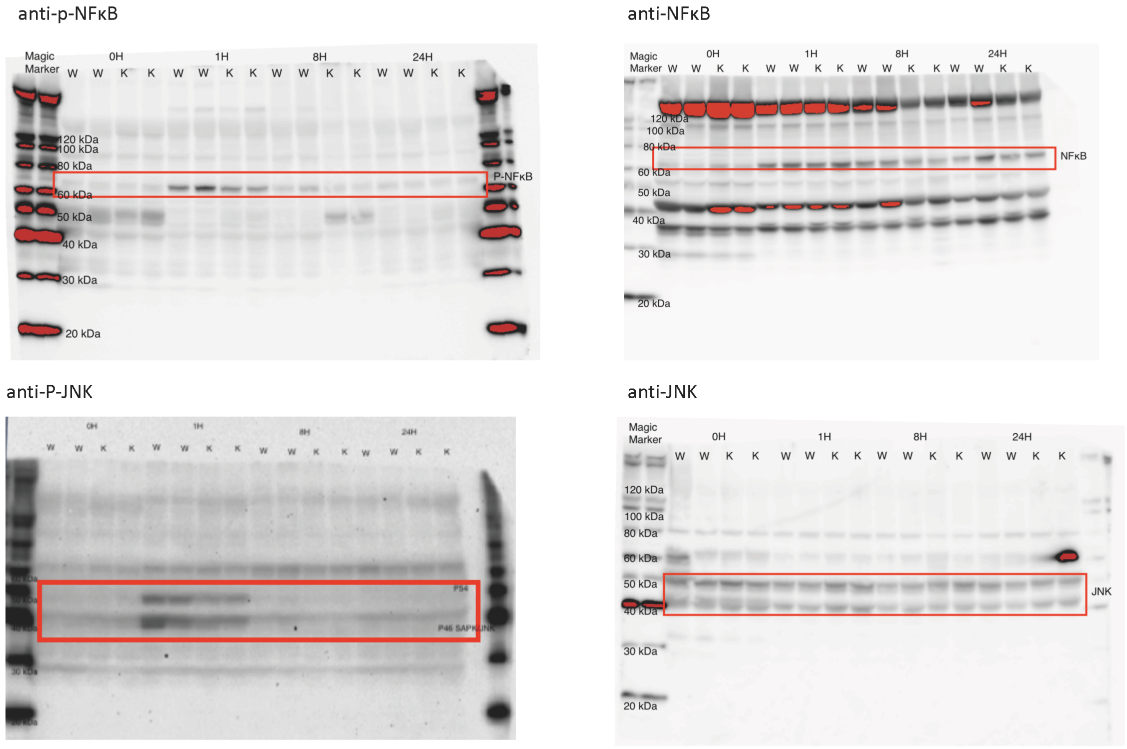

Supplemental Table 1. Genes involved in immune system process.

Gene ID	Gene Name
NM_145227	2'-5' oligoadenylate synthetase 2(Oas2)
NM_145226	2'-5' oligoadenylate synthetase 3(Oas3)
NM_145209	2'-5' oligoadenylate synthetase-like 1(Oasl1)
NM_011854	2'-5' oligoadenylate synthetase-like 2(Oasl2)
NM_001252469	BCL2-associated athanogene 6(Bag6)
NM_020001	C-type lectin domain family 4, member n(Clec4n)
NM_145437	CD300 molecule like family member d(Cd300ld)
NM_134158	CD300C molecule 2(Cd300c2)
NM_009690	CD5 antigen-like(Cd5l)
NM_010016	CD55 molecule, decay accelerating factor for complement(Cd55)
NM_001042605	CD74 antigen (invariant polypeptide of major histocompatibility complex, class II antigen-associated)(Cd74)
NM_172689	DEAD (Asp-Glu-Ala-Asp) box polypeptide 58(Ddx58)
NM_030150	DEXH (Asp-Glu-X-His) box polypeptide 58(Dhx58)
NM_010846	MX dynamin-like GTPase 1(Mx1)
NM_013606	MX dynamin-like GTPase 2(Mx2)
NM_001033207	NLR family, CARD domain containing 5(Nlrc5)
AK078327	OTU domain containing 7B(Otud7b)
NM_175397	Sp110 nuclear body protein(Sp110)
NM_001139519	Z-DNA binding protein 1(Zbp1)
NM_010130	adhesion G protein-coupled receptor E1(Adgre1)
NM_010730	annexin A1(Anxa1)
NM_009735	beta-2 microglobulin(B2m)
NM_007609	caspase 4, apoptosis-related cysteine peptidase(Casp4)
NM_007574	complement component 1, q subcomponent, C chain(C1qc)
NM_007572	complement component 1, q subcomponent, alpha polypeptide(C1qa)
NM_009777	complement component 1, q subcomponent, beta polypeptide(C1qb)
NM_013484	complement component 2 (within H-2S)(C2)
NM_008823	complement factor properdin(Cfp)
NM_007995	ficolin A(Fcna)
NM_001172117	hemopoietic cell kinase(Hck)
NM_010380	histocompatibility 2, D region locus 1(H2-D1)
NM_001267808	histocompatibility 2, D region locus L(H2-L)
NM_001001892	histocompatibility 2, K1, K region(H2-K1)
NM_010394	histocompatibility 2, Q region locus 7(H2-Q7)
NM_010398	histocompatibility 2, T region locus 23(H2-T23)
NM_025378	interferon induced transmembrane protein 3(Ifitm3)
NM_027835	interferon induced with helicase C domain 1(Ifih1)
NM_016850	interferon regulatory factor 7(Irf7)
NM_008331	interferon-induced protein with tetratricopeptide repeats 1(Ifit1)
NM_010501	interferon-induced protein with tetratricopeptide repeats 3(Ifit3)
NM_001291894	leukocyte immunoglobulin-like receptor, subfamily B, member 4A(Lilrb4a)
NM_008491	lipocalin 2(Lcn2)
NM_010766	macrophage receptor with collagenous structure(Marco)
NM_178440	myosin IG(Myo1g)
NM_011095	paired Ig-like receptor B(Pirb)
NM_021384	radical S-adenosyl methionine domain containing 2(Rsad2)

Supplemental Table 2. Genes involved in mitotic nuclear division.

Gene ID	Gene Name
NM_009772	BUB1, mitotic checkpoint serine/threonine kinase(Bub1)
NM_025995	F-box protein 5(Fbxo5)
NM_023294	NDC80 kinetochore complex component(Ndc80)
NM_023284	NUF2, NDC80 kinetochore complex component(Nuf2)
NM_026282	SPC24, NDC80 kinetochore complex component, homolog (S. cerevisiae)(Spc24)
NM_001305800	SPC25, NDC80 kinetochore complex component, homolog (S. cerevisiae)(Spc25)
NM_028109	TPX2, microtubule-associated(Tpx2)
AK147951	anaphase-promoting complex subunit 5(Anapc5)
NM_028390	anillin, actin binding protein(Anln)
NM_009791	asp (abnormal spindle)-like, microcephaly associated (Drosophila)(Aspm)
NM_011497	aurora kinase A(Aurka)
NM_009689, NM_001012273	baculoviral IAP repeat-containing 5(Birc5)
NM_023223	cell division cycle 20(Cdc20)
NM_011799	cell division cycle 6(Cdc6)
NM_175384, NM_001110162	cell division cycle associated 2(Cdca2)
NM_013538	cell division cycle associated 3(Cdca3)
NM_026410	cell division cycle associated 5(Cdca5)
NM_026560	cell division cycle associated 8(Cdca8)
NM_173762	centromere protein E(Cenpe)
NM_028131	centromere protein N(Cenpn)
NM_009828	cyclin A2(Ccna2)
NM_172301	cyclin B1(Ccnb1)
NM_007630	cyclin B2(Ccnb2)
NM_007659	cyclin-dependent kinase 1(Cdk1)
NM_001014976	extra spindle pole bodies 1, separase(Espl1)
NM_010615	kinesin family member 11(Kif11)
NM_183046	kinesin family member 20B(Kif20b)
NM_024245	kinesin family member 23(Kif23)
NM_001290662	kinesin family member 2C(Kif2c)
NM_001042421	kinetochore associated 1(Kntc1)
NM_029617	kinetochore scaffold 1(Knl1)
NM_026412	kinetochore-localized astrin/SPAG5 binding(Knstrn)
NM_146171	non-SMC condensin I complex, subunit D2(Ncapd2)
NM_133762	non-SMC condensin II complex, subunit G2(Ncapg2)
NM_133851	nucleolar and spindle associated protein 1(Nusap1)
NM_011121	polo-like kinase 1(Plk1)
AK048254	retinoblastoma binding protein 8(Rbbp8)
NM_028232	shugoshin-like 1 (S. pombe)(Sgol1)
NM_025581	spindle and kinetochore associated complex subunit 1(Ska1)
NM_001301412	structural maintenance of chromosomes 2(Smc2)
NM_133786	structural maintenance of chromosomes 4(Smc4)
NM_026785	ubiquitin-conjugating enzyme E2C(Ube2c)
NM_026507	zwilch kinetochore protein(Zwilch)

Supplemental Table 3. Reagents used in this study.

**Reagents**	**Final Conc**	**Source**	**Material Number**	**Location**
MitoTracker Red CMXRos	250 nM	Molecular Probes	M7512	(Rockford, IL, USA)
MitoTracker Green FM	25 nM	Molecular Probes	M7514	(Rockford, IL, USA)
Hoechst 33342	1 µg/ml	Dojindo	H342	(Kumamoto, Japan)
DAPI	5 µg/ml	Dojindo	D523	(Kumamoto, Japan)
LPS from *Escherichia coli* O111:B4	100 ng/ml	Sigma-aldrich	L2630	(St. Louis, MO, USA)
Tetramethylrhodamine, Ethyl Ester, Perchlorate (TMRE)	100 nM	Molecular Probes	T669	(Rockford, IL, USA)
Tauroursodeoxycholic acid	500 µM / 5 mM	Tcichemicals	T1567	(Tokyo, Japan)
Thapsigargin	2 µM / 5 µM	Sigma-aldrich	T9033	(St. Louis, MO, USA)
7AAD 5 µl (0.25 µg)/test (1x10^6 cells)		BD Biosciences	559925	(Sparks, MD, USA)
ApopTag® Peroxidase In Situ Apoptosis Detection Kit		Milipore	S7100	(Burlington, Boston, USA)
Cellstain- Trypan Blue		Dojindo	T375	(Kumamoto, Japan)

Supplemental Table 4. Antibodies used in this study.

Rat monoclonal anti F4/80 (1:200 dilution; AbD serotec, Hercules, CA, USA)
Goat polyclonal anti-IL-1β (1:1000 dilution for western and 1:200 dilution for immunostain; Sigma-aldrich, St. Louis, MO, USA)
Rabbit polyclonal anti-LC3A/B (1:1000 dilution for western and 1:200 dilution for immunostain; Cell signaling, Danvers, MA, USA)
Mouse monoclonal anti-OPA1 (1:200 dilution ; BD Biosciences, Sparks, MD, USA)
Rat monoclonal anti Ly6G (1:200 dilution; BD Biosciences, Sparks, MD, USA)
Rat monoclonal anti-CD3 (1:200 dilution; AbD serotec, Hercules, CA, USA)
Rat monoclonal anti-CD45R/B220, clone RA3-6B2 (1:200 dilution; BioLegend, San Diego, CA, USA)
Rabbit polyclonal anti- pNFκB, p65 subunit (Ser536) (1:1000 dilution; Cell signaling, Danvers, MA, USA)
Mouse monoclonal anti-NFκB, p65 subunit, clone 12H11 (1:1000 dilution; Chemicon, Temecula, CA, USA)
Mouse monoclonal anti-NLRP3/NALP3 (Cryo-2) (1:1000 dilution; Adipogen, San Diego, CA, USA)
Mouse monoclonal anti-DLP1(DRP1) (1:200 dilution ; BD Biosciences, Sparks, MD, USA)
Phospho DRP1 (Ser616) antibody (1:1000 dilution; Cell signaling, Danvers, MA, USA)
Phospho DRP1 (Ser637) antibody (1:1000 dilution; Cell signaling, Danvers, MA, USA)
Rabbit polyclonal anti-Caspase 3 (1:1000 dilution; Cell signaling, Danvers, MA, USA)
Rabbit polyclonal anti-SQSTM1/p62 (1:1000 dilution; Cell signaling, Danvers, MA, USA)
Rabbit polyclonal anti-PINK1 (1:1000 dilution; Cell signaling, Danvers, MA, USA)
Rabbit polyclonal anti-phospho-JNK (Thr183/Tyr185) (1:1000 dilution; Cell signaling, Danvers, MA, USA)
Mouse monoclonal anti-JNK (1:1000 dilution; Santa Cruz Biotechnology, Santa Cruz, CA, USA)
Rabbit monoclonal anti-phospho-eIF2α (Ser51) (1:1000 dilution; Cell signaling, Danvers, MA, USA)
Rabbit monoclonal anti-eIF2α (1:1000 dilution; Santa Cruz Biotechnology, Santa Cruz, CA, USA)
Horse anti-mouse IgG, HRP-linked antibody (1:5000 dilution; Cell signaling, Danvers, MA, USA)
Goat anti-rabbit IgG, HRP-linked antibody (1:5000 dilution; Cell signaling, Danvers, MA, USA)
Goat anti-rat IgG, HRP-linked antibody (1:5000 dilution; Cell signaling, Danvers, MA, USA)
Donkey anti-goat IgG, HRP-linked antibody (1:1000 dilution; Santa Cruz Biotechnology, Santa Cruz, CA, USA)
Rabbit monoclonal anti-GAPDH (HRP Conjugate) (1:5000 dilution; Cell signaling, Danvers, MA)
PE-Cy™7 Rat anti-mouse CD45 (1:100 dilution ; BD Biosciences, Sparks, MD, USA)
APC-Cy™7 Rat anti-Mouse CD11b (1:100 dilution ; BD Biosciences, Sparks, MD, USA)
FITC rat anti-mouse F4/80 (1:100 dilution; BioLegend, San Diego, CA, USA)
APC rat anti-mouse CD206 (MMR) (1:100 dilution; BioLegend, San Diego, CA, USA)
APC anti-mouse CD64 (1:100 dilution; BioLegend, San Diego, CA, USA)
PE anti-mouse CD80 (1:100 dilution; BioLegend, San Diego, CA, USA)
Alexa Fluor® 488 donkey anti-rabbit IgG (1:200 dilution; Thermo Fisher Scientific, Rockford, IL, USA)
Alexa Fluor® 594 chicken anti-goat IgG(H+L) (1:200 dilution; Thermo Fisher Scientific, Rockford, IL, USA)
Alexa Fluor® 488 rabbit anti-rat IgG (H+L) (1:200 dilution; Thermo Fisher Scientific, Rockford, IL, USA)
Alexa Fluor® 594 goat anti-mouse IgG(H+L) (1:200 dilution; Thermo Fisher Scientific, Rockford, IL, USA)

Supplemental Table 5. Real-time PCR primers used in this study.

Gene	Forward primer (5' – 3')	Reverse primer (5' – 3')	Product size (bp)
Tnfa	CCACCACGCTCTTCTGTCTA	AGGGTCTGGGCCATAGAACT	103
Mcp1	GCAGTTAACGCCCCACTCA	CCAGCCTACTCATTGGGATCA	67
Chop	AGCCTGGTATGAGGATCTGC	CTCCTGCTCCTTCTCCTTCA	319
P8	CAGACCACAGACACCACACC	TCTGCTTCTTGCTCCCATCT	222
Trib3	CCCACAGGCACAGAGTACAC	CGTCCTCTCACAGTTGCTGA	324
Atf3	ACCTCCTGGGTCACTGGTATTTG	TTCTTTCTCGCCGCCTCCTTTTCC	215
Il6	CCGGAGAGGAGACTTCACAG	TTCTGCAAGTGCATCATCGT	166
Il-1b	TGTGAAATGCCACCTTTTGA	GGTCAAAGGTTTGGAAGCAG	94
Ifnb1	CCCTATGGAGATGACGGAGA	CTGTCTGCTGGTGGAGTTCA	161
Drp1	TGCCTCAGATCGTCGTAGTG	TGACCACACCAGTTCCTCTG	101
Gapdh	CCATCACTGCCACCCAGAAG	GATGCAGGGATGATGTTC	91
