# Supplementary material for: Dynamin-related protein 1 deficiency accelerates lipopolysaccharide-induced acute liver injury and inflammation in mice
Source: Commun Biol. 2021 Jul 21;4:894. doi: 10.1038/s42003-021-02413-6 (PMC8295278; doi:10.1038/s42003-021-02413-6)
Supplement: Supplementary file 3 — Reporting Summary [file 42003_2021_2413_MOESM3_ESM.pdf]

nature research

Corresponding author(s): Lixiang Wang & Masatoshi Nomura

Last updated by author(s): Jun 24, 2021

Reporting Summary

Nature Research wishes to improve the reproducibility of the work that we publish. This form provides structure for consistency and transparency in reporting. For further information on Nature Research policies, see our Editorial Policies and the Editorial Policy Checklist.

Statistics

For all statistical analyses, confirm that the following items are present in the figure legend, table legend, main text, or Methods section.

n/a	Confirmed	
☐	☒	The exact sample size (*n*) for each experimental group/condition, given as a discrete number and unit of measurement
☐	☒	A statement on whether measurements were taken from distinct samples or whether the same sample was measured repeatedly
☐	☒	The statistical test(s) used AND whether they are one- or two-sided<br>*Only common tests should be described solely by name; describe more complex techniques in the Methods section.*
☐	☒	A description of all covariates tested
☐	☒	A description of any assumptions or corrections, such as tests of normality and adjustment for multiple comparisons
☐	☒	A full description of the statistical parameters including central tendency (e.g. means) or other basic estimates (e.g. regression coefficient) AND variation (e.g. standard deviation) or associated estimates of uncertainty (e.g. confidence intervals)
☐	☒	For null hypothesis testing, the test statistic (e.g. *F*, *t*, *r*) with confidence intervals, effect sizes, degrees of freedom and *P* value noted<br>*Give P values as exact values whenever suitable.*
☒	☐	For Bayesian analysis, information on the choice of priors and Markov chain Monte Carlo settings
☒	☐	For hierarchical and complex designs, identification of the appropriate level for tests and full reporting of outcomes
☒	☐	Estimates of effect sizes (e.g. Cohen's *d*, Pearson's *r*), indicating how they were calculated

Our web collection on statistics for biologists contains articles on many of the points above.

Software and code

Policy information about availability of computer code

Data collection	For microarray analysis, cRNA was hybridized to a 60K 60-mer oligomicroarray (SurePrint G3 Mouse Gene Expression Microarray 8x60K v2 ; Agilent Technologies), and the hybridized microarray slides were scanned using an Agilent scanner. The tissue sections were analyzed under a BZ-8000 microscope (Keyence, Osaka, Japan) or a confocal microscope LSM700 (Zeiss, Oberkochen, Germany). ALT and AST levels were collected by using the DRI-CHEM3500 Chemistry Analyzer (Fujifilm, Tokyo, Japan). The levels of cytokines were detected using the BD Cytometric Bead Array (CBA) Cytokine kit (Becton-Dickinson, Franklin Lakes, NJ, USA) and a NovoCyte flow cytometer (ACEA Biosciences, San Diego, CA, USA).
Data analysis	Confocal microscope images were analyzed using ZEN software (Zeiss, Oberkochen, Germany). The levels of cytokines were analyzed using a NovoCyte flow cytometer NovoExpress software. For microarray, the relative hybridization intensities and background hybridization values were calculated using Feature Extraction Software version 9.5.1.1 (Agilent Technologies). Two-tailed Student's t-test was performed to compare two groups using Microsoft Excel (Mac 201012 version 16.16.27; Microsoft Japan, Tokyo, Japan). Two-way analysis of variance (ANOVA) with Bonferroni's Post Hoc Test or Tukey's post hoc test or ordinary one-way ANOVA was performed to compare multiple groups using GraphPad Prism 6.0 software (GraphPad, San Diego, CA, USA).

For manuscripts utilizing custom algorithms or software that are central to the research but not yet described in published literature, software must be made available to editors and reviewers. We strongly encourage code deposition in a community repository (e.g. GitHub). See the Nature Research guidelines for submitting code & software for further information.

Data

Policy information about availability of data

All manuscripts must include a data availability statement. This statement should provide the following information, where applicable:

- Accession codes, unique identifiers, or web links for publicly available datasets
- A list of figures that have associated raw data
- A description of any restrictions on data availability

The data that support the findings of this study are available from the corresponding authors upon reasonable request. The microarray data from this publication have been submitted to the Gene Expression Omnibus database [http://www.ncbi.nlm.nih.gov/geo/] and assigned an identifier [accession: GSE156982].

Field-specific reporting

Please select the one below that is the best fit for your research. If you are not sure, read the appropriate sections before making your selection.

- [x] Life sciences
- [ ] Behavioural & social sciences
- [ ] Ecological, evolutionary & environmental sciences

For a reference copy of the document with all sections, see nature.com/documents/nr-reporting-summary-flat.pdf

Life sciences study design

All studies must disclose on these points even when the disclosure is negative.

Sample size	The sample size was chosen on the basis of prior studies that showed significant effects with similar sample sizes.
Data exclusions	Data were not excluded from analysis.
Replication	Replicated experiments were successful and support conclusions drawn in this report.
Randomization	Samples were chosen randomly from each genotype per treatment per time point.
Blinding	Investigators were not blinded during experiments.

Reporting for specific materials, systems and methods

We require information from authors about some types of materials, experimental systems and methods used in many studies. Here, indicate whether each material, system or method listed is relevant to your study. If you are not sure if a list item applies to your research, read the appropriate section before selecting a response.

Materials & experimental systems

n/a	Involved in the study	
[ ]	[x]	Antibodies
[ ]	[x]	Eukaryotic cell lines
[x]	[ ]	Palaeontology and archaeology
[ ]	[x]	Animals and other organisms
[x]	[ ]	Human research participants
[x]	[ ]	Clinical data
[x]	[ ]	Dual use research of concern

Methods

n/a	Involved in the study	
[x]	[ ]	ChIP-seq
[ ]	[x]	Flow cytometry
[x]	[ ]	MRI-based neuroimaging

Antibodies

Antibodies used

Rat monoclonal anti F4/80 (1:200 dilution; AbD serotec, Hercules, CA, USA)
Rabbit polyclonal anti-Caspase 3 (1:1000 dilution; Cell signaling, Danvers, MA, USA)
Phospho DRP1 (Ser616) antibody (1:1000 dilution; Cell signaling, Danvers, MA, USA)
Phospho DRP1 (Ser637) antibody (1:1000 dilution; Cell signaling, Danvers, MA, USA)
Mouse monoclonal anti-DLP1(DRP1) (1:200 dilution ; BD Biosciences, Sparks, MD, USA)
Goat polyclonal anti-IL-1β (1:1000 dilution for western and 1:200 dilution for immunostain; Sigma-aldrich, St. Louis, MO, USA)
Rabbit polyclonal anti-LC3A/B (1:1000 dilution for western and 1:200 dilution for immunostain; Cell signaling, Danvers, MA, USA)
Mouse monoclonal anti-OPA1 (1:200 dilution ; BD Biosciences, Sparks, MD, USA)
Rat monoclonal anti Ly6G (1:200 dilution; BD Biosciences, Sparks, MD, USA)
Rat monoclonal anti-CD3 (1:200 dilution; AbD serotec, Hercules, CA, USA)

Rat monoclonal anti-CD45R/B220, clone RA3-6B2 (1:200 dilution; BioLegend, San Diego, CA, USA)
Rabbit polyclonal anti- pNFκ B, p65 subunit (Ser536) (1:1000 dilution; Cell signaling, Danvers, MA, USA)
Mouse monoclonal anti-NFκ B, p65 subunit, clone 12H11 (1:1000 dilution; Chemicon, Temecula, CA, USA)
Mouse monoclonal anti-NLRP3/NALP3 (Cryo-2) (1:1000 dilution; Adipogen, San Diego, CA, USA)
Rabbit polyclonal anti-phospho mTOR (Ser2448) (1:1000 dilution; Cell signaling, Danvers, MA, USA)
Rabbit polyclonal anti-mTOR (1:1000 dilution; Cell signaling, Danvers, MA, USA)
Rabbit polyclonal anti-SQSTM1/p62 (1:1000 dilution; Cell signaling, Danvers, MA, USA)
Rabbit polyclonal anti-PINK1 (1:1000 dilution; Cell signaling, Danvers, MA, USA)
Rabbit polyclonal anti-phospho-JNK (Thr183/Tyr185) (1:1000 dilution; Cell signaling, Danvers, MA, USA)
Mouse monoclonal anti-JNK (1:1000 dilution; Santa Cruz Biotechnology, Santa Cruz, CA, USA)
Rabbit monoclonal anti-phospho-eIF2α (Ser51) (1:1000 dilution; Cell signaling, Danvers, MA, USA)
Rabbit monoclonal anti-eIF2α (1:1000 dilution; Santa Cruz Biotechnology, Santa Cruz, CA, USA)
Horse anti-mouse IgG, HRP-linked antibody (1:5000 dilution; Cell signaling, Danvers, MA, USA)
Goat anti-rabbit IgG, HRP-linked antibody (1:5000 dilution; Cell signaling, Danvers, MA, USA)
Goat anti-rat IgG, HRP-linked antibody (1:5000 dilution; Cell signaling, Danvers, MA, USA)
Donkey anti-goat IgG, HRP-linked antibody (1:1000 dilution; Santa Cruz Biotechnology, Santa Cruz, CA, USA)
Rabbit monoclonal anti-GAPDH (HRP Conjugate) (1:5000 dilution; Cell signaling, Danvers, MA)
PE-Cy™7 Rat anti-mouse CD45 (1:100 dilution ; BD Biosciences, Sparks, MD, USA)
APC-Cy™7 Rat anti-Mouse CD11b (1:100 dilution ; BD Biosciences, Sparks, MD, USA)
FITC rat anti-mouse F4/80 (1:100 dilution; BioLegend, San Diego, CA, USA)
APC rat anti-mouse CD206 (MMR) (1:100 dilution; BioLegend, San Diego, CA, USA)
APC anti-mouse CD64 (1:100 dilution; BioLegend, San Diego, CA, USA)
PE anti-mouse CD80 (1:100 dilution; BioLegend, San Diego, CA, USA
Alexa Fluor® 488 donkey anti-rabbit IgG (1:200 dilution; Thermo Fisher Scientific, Rockford, IL, USA)
Alexa Fluor® 594 chicken anti-goat IgG(H+L) (1:200 dilution; Thermo Fisher Scientific, Rockford, IL, USA)
Alexa Fluor® 488 rabbit anti-rat IgG (H+L) (1:200 dilution; Thermo Fisher Scientific, Rockford, IL, USA)
Alexa Fluor® 594 goat anti-mouse IgG(H+L) (1:200 dilution; Thermo Fisher Scientific, Rockford, IL, USA)

Validation

Antibodies have been validated either by the suppliers or by our previous work (Wang et al, 2015),

Eukaryotic cell lines

Policy information about cell lines

Cell line source(s)

Mouse primary hepatocyte and hepatic nonparenchymal cell were isolated as previously described (Severgnini, M. et al, 2012).

Authentication

None of the cell lines have been authenticated.

Mycoplasma contamination

Cell lines were not tested for mycoplasma contamination but no indication of contamination was observed.

Commonly misidentified lines
(See ICLAC register)

No commonly misidentified cell lines were used.

Animals and other organisms

Policy information about studies involving animals; ARRIVE guidelines recommended for reporting animal research

Laboratory animals

We purchased 8–12-week-old male C57BL/6J mice from KBT Oriental Co., Ltd. (Saga, Japan). We created Drp1LiKO (Alb-CreTg/+ Drp1flox/flox) and control (Drp1flox/flox, sibling littermates) mice and genotyped them for the Drp1 conditional allele and the Cre transgene using PCR, as previously described (Wang et al. 2015)

Wild animals

No wild animals were used in this study.

Field-collected samples

The mice were maintained in a standard specific-pathogen free room at room temperature (22°C–24°C) and 50%-60% relative humidity under a 12 h/12 h light/dark cycle (lights off at 8:00 p.m.). The mice were fed a normal chow diet (NCD; 5.4% fat, CRF-1; Orient Yeast, Tokyo, Japan) ad libitum. Experiments were performed between 10:00 and 11:00 a.m. after overnight fasting, except the LPS challenge and primary hepatocyte isolation (ad libitum).

Ethics oversight

All mouse procedures and protocols were approved by the Ethics Committees on Animal Experimentation (Kyushu University, Graduate School of Medicine, Japan) and performed in accordance with the Guide for the Care and Use of Laboratory Animals.

Note that full information on the approval of the study protocol must also be provided in the manuscript.

Flow Cytometry

Plots

Confirm that:

- [x] The axis labels state the marker and fluorochrome used (e.g. CD4-FITC).
- [x] The axis scales are clearly visible. Include numbers along axes only for bottom left plot of group (a 'group' is an analysis of identical markers).
- [x] All plots are contour plots with outliers or pseudocolor plots.
- [x] A numerical value for number of cells or percentage (with statistics) is provided.

Methodology

Sample preparation	LPS was injected intraperitoneally into mice at 5 mg/kg body weight, and liver and serum samples were collected from the inferior vena cava at different time points (0, 1, 4, 8, 24, and 48 h). The liver tissue was perfused with cold PBS through the portal vein and then chopped into 1-2 mm pieces. Next, we added 100 mg of liver tissue to 1 mL of cell lysis buffer (R&D System, Minneapolis, MN, USA) and homogenized it using a tissue homogenizer. Finally, we diluted liver, serum, and cell culture supernatant samples with known high protein concentrations at 1:50 using an assay diluent and quantified the levels of various types of cytokines .
Instrument	NovoCyte flow cytometer (ACEA Biosciences, San Diego, CA, USA)
Software	NovoExpress software
Cell population abundance	No post-sort fractions were collected.
Gating strategy	Before analysis of fluorescence, live, single cells were gated using forward and side scatter parameters (FSC, SSC) to exclude cell debris and doublets. Gating strategy was showed in Figure 3. Pro-inflammatory (M1) macrophages were identified as 7AAD-CD45+CD11b+F4/80+CD80High CD64Low, 7AAD-CD45+CD11b+ F4/80+CD80Low CD64High, and 7AAD-CD45+CD11b+ F4/80+CD80High CD64High; anti-inflammatory (M2) macrophages were identified as 7AAD-CD45+CD11b+ F4/80+CD206High macrophages, respectively.

- [x] Tick this box to confirm that a figure exemplifying the gating strategy is provided in the Supplementary Information.
