# Supplementary material for: Dynamin-related protein 1 deficiency accelerates lipopolysaccharide-induced acute liver injury and inflammation in mice
Source: Commun Biol. 2021 Jul 21;4:894. doi: 10.1038/s42003-021-02413-6 (PMC8295278; doi:10.1038/s42003-021-02413-6)
Supplement: Supplementary file 5 — Description of Additional Supplementary Files [file 42003_2021_2413_MOESM5_ESM.pdf]

Description of Additional Supplementary Files

File name: Supplementary data 1

Description: Source data for graphs and charts
